# Extracellular Vesicles: From Biomarkers to Therapeutic Tools

**DOI:** 10.3390/biology9090258

**Published:** 2020-08-31

**Authors:** Simona Bernardi, Carolina Balbi

**Affiliations:** 1 Department of Clinical and Experimental Sciences, University of Brescia, Bone Marrow Transplant Unit, ASST Spedali Civili, 25123 Brescia, Italy; 2 Centro di Ricerca Emato-Oncologica AIL (CREA), ASST Spedali Civili, 25123 Brescia, Italy; 3 Cellular & Molecular Cardiology Laboratory, Cardiocentro Ticino, Associated Institute of University of Zurich, 6900 Lugano, Switzerland; carolina.balbi@cardiocentro.org

**Keywords:** extracellular vesicles, exosomes, biomarkers, drug carrier, liquid biopsy, extracellular vesicle engineering and isolation

## Abstract

Intercellular communication is an essential hallmark of multicellular organisms and can be mediated through direct cell–cell contact or transfer of secreted molecules. In the last two decades, a third mechanism for intercellular communication has emerged that involves intercellular transfer of extracellular vesicles (EVs). EVs are membranous vesicles of 30–5000 nm in size. Based on their dimension and biogenesis, EVs can be divided into different categories, such as microvesicles, apoptotic bodies, ectosomes, and exosomes. It has already been demonstrated that protein changes, expressed on the surfaces or in the content of these vesicles, may reflect the status of producing cells. For this reason, EVs, and exosomes in particular, are considered ideal biomarkers in several types of disease—from cancer diagnosis to heart rejection. This aspect opens different opportunities in EVs clinical application, considering the importance given to liquid biopsy in the recent years. Furthermore, extracellular vesicles can be natural or engineered carriers of cytoprotective or cytotoxic factors and applied, as a therapeutic tool, from regenerative medicine to target cancer therapy. This is of pivotal importance in the so called “era of the 4P medicine”. This Editorial focuses on recent findings pertaining to EVs in different medical areas, from biomarkers to therapeutic applications.

## 1. Introduction

Intercellular communication is essential in multicellular organisms and can be mediated through direct cell–cell contact or transfer of secreted molecules [1]. In the last two decades, a third mechanism for intercellular communication, that involves the intercellular transfer of extracellular vesicles (EVs), has emerged [2]. EVs are membrane vesicles of 30–1000 nm in size. They can be isolated in vitro from cell-conditioned medium, as well as from different body fluids (e.g., such as plasma, urine, breast milk) [3]. Based on their dimension EVs can be divided into different categories: small vesicles, such as exosome (<200 nm), and large vesicles, such as microvesicles (MVs), ectosomes/oncosomes (200–1000 nm) [4]. EVs are also categorized based on their biogenesis. EVs can be released either through the outward budding of the plasma membrane, termed shedding microvesicles (MVs), or ectosomes [5]. The other mechanism involves the inward budding of the endosomal membrane, resulting in the formation of multivesicular bodies (MVBs), with exosomes released by fusion of the outer MVB membrane to the plasma membrane [6].

EVs are loaded with a diverse range of proteins, some of which are common to most EVs subsets released from most cell types, such as the membrane-bound tetraspanins CD9, CD81, and CD63 [4]. Others, instead, are detected in EVs derived from only a specific subset of cell types. This is due to their capability of carrying different markers from the cell of origin. For such reasons EVs have been recently pointed out as one of the main characters for liquid biopsy (biomarkers) and therapeutic application.

## 2. EVs as Biomarkers

These shuttled markers may be proteins, as well as nucleic acids and lipids, and are sheltered and conserved in their original stability, structure and sequence [7]. Moreover, EVs cargoes have been analyzed and characterized from many sources, with their identities used to investigate the biogenesis of the vesicles and to develop diagnostic biomarker panels for human disease [8]. In fact, the circulating EVs, both in peripheral blood and other biological fluids, such as cerebrospinal fluid and urine [9,10], are dynamically monitorable and reflect the physiological and pathophysiological characteristics of the parental cell. In addition, their concentration in biological fluids seems to be disease-state dependent [11,12,13]. Thanks to these features, EVs, and exosomes in particular, have been identified as reliable biomarkers for diagnostic and minimal residual disease monitoring purposes. Moreover, their prognostic impacts have been demonstrated in different clinical settings, such as cardiac [14], neurological and neurodegenerative [15,16,17], immune and autoimmune [18,19], oncological [20] and onco-hematological diseases [11,21], and pregnancy complications [22]. Nevertheless, the isolation and characterization of EVs and exosomes’ cargoes remained a big challenge for years because of the low quantity. In particular, the challenge of protein cargo identification from exosomes preparations was the lack of amplification techniques compared to nucleic acids investigation. However, thanks to rapid improvements in proteomic technologies, such as mass spectrometry [23] and Raman technology [12,23], EVs protein characterization is not only feasible, but of great interest from a biological and medical point of view.

Regarding their isolation, the possibility to isolate a specific subset of exosomes or EVs released by a specific cell population is of pivotal importance in order to increase the sensitivity of the markers detection, and to improve the specificity of the investigation [24,25]. In recent years, different approaches have been described and compared with ultra-centrifuge or conventional isolation kits. In particular, many efforts have been focused on the immuno-selection of exosomes based on the binding of specific antigens expressed on their membrane [25] or on peptide affinity. These methods normally involve antibodies that are either conjugated to beads (i.e., magnetic) or conjugated with a secondary antibody bead. This aspect presents a particular impact in oncology and onco-hematology diseases because it enables the scientists to detect active malignant cells [19,26,27], overcoming the need of their localization. In addition, the isolation of a specific EVs population released by pathogenic cells seems of great interest in the screening of people considered under risk of development of particular diseases [28,29]. Moreover, the feasibility of the isolation of a specific exosomes or vesicles population opens the opportunity to set up effective liquid biopsy approaches, in order to reduce invasive and painful conventional tissue biopsies [30,31,32]—in particular for the very challenging anatomical districts such as the brain [33,34]. In fact, in diseases affecting the central nervous system, neurologists have no access to the diseased tissue, with the exception of extreme cases requiring a cerebral biopsy. Therefore, EVs released by neural and non-neural cells are considered as possible vehicles of both clinical and biological information. In addition to the anatomical challenge, central nervous system diseases are disadvantaged by the very reduced amount of EVs released by neural cells. Therefore, brain specific EVs are very diluted, if present, in peripheral biological fluids, increasing the limit of EVs biomarkers detection by current available technologies [35]. In this scenario, immuno-selection of exosomes seems the most suitable approach in order to overcome this limit. These approaches have been explored in different studies focused on central nervous system disease. EVs markers for extra-cranial (CD62E+) and intra-cranial (CD31+CD42b−PS+) localization of the stenosis have been recognized in the case of stroke and stroke mimics. Other specific markers useful for the isolation of EVs released by neural cell populations are L1CAM and CD171. The isolation of EVs exposing these markers on their surface opened the possibility to better estimate the time of stroke and the clinical severity. In the future, the routinely available and standardized detection techniques for specific EVs sub-populations may result as an additional tool to improve therapeutic strategies, or to better stratify the patients’ risk [36,37]. Additionally, in brain tumors, the feasibility of overcoming a conventional tissue biopsy and of following disease progression through EVs is based on the detection limits. Up to now, some central nervous system tumor-EVs specific markers have already been identified, and they may help the set-up of valuable liquid biopsy approaches in future clinical neurology [38].

Therefore, the synergy between the isolation and enrichment strategies and the new technologies for the analysis of proteins and nucleic acids, such as proteomics and genomics, are increasing the present clinical application of the EVs and exosomes as reliable biomarkers, and are the bases for the future.

## 3. EVs as Therapeutic Tools

The capability of these vesicles to carry messages and information to target cells inspired the scientists in using exosomes and EVs as potential shutters for drug delivery. Compared to the synthetic carriers, such as LNPs or polymeric micelles, that present toxicity problems, EVs are safe. Furthermore, EVs possess the unique ability to cross tissue and cellular barriers [39]. Loading of molecules into extracellular vesicles is a strategy that combines the physiological activity of the extracellular vesicles with the apported modification. For example, modification of EVs from mesenchymal stem cells (MSCs) will result in a double effect: the inhibitory effects on immune responses exerted by MSCs derived EVs [40] plus the drug activity. This type of strategy was successfully used in a mouse model of pancreatic cancer. EVs derived from MSCs and loaded with high amounts of anti-KRASG12D siRNA showed a significant increase in mouse survival [41].

EVs can also be engineered to target specific organs. Indeed, the fate of this carrier, when not locally injected, remains elusive. Mentkowski et al., for example, modified cardiosphere derived cells in order to express Lamp2b, an exosome membrane protein, fused to a cardiomyocyte specific peptide (CMP). Such a stratagem increased retention of the exosome by cardiomyocyte, compared to the naive one, and resulted in higher cardioprotection activity [42].

As suggested previously, EVs derived from stem or progenitor cells can also be used unmodified, thanks to their ability to deliver exogenous therapeutic cargo [43].

The evidence that extracellular vesicles secreted by certain cells could be used for therapeutic application, especially in the regenerative medicine field, derives from previous studies on cell therapy. For example, in the cardiovascular field, researchers initially thought that the cardioprotective properties of mesenchymal stem cells (MSCs), injected into the injured heart, resulted from their differentiation into healthy myocardium [44]. However, different studies later showed that such effects were due to the paracrine activity exploited by MSCs, and in particular from the vesicular part [45]. Furthermore, EVs possess numerous advantages over cell-based therapies in the context of regenerative medicine, such as less limitation related to safety and feasibility of canonical cell transplantation like cell engraftment, survival and immunocompatibility. Moreover, EVs can be easily stored and transported for long periods. Additionally, the injection of EVs does not present risk of tumor generation because they are unable to replicate.

The current “state of the art” of extracellular vesicles fractions used as a therapeutic agent has presented different active clinical trials. Phase-1 and phase-2 clinical trials (ClinicalTrials.gov Identifier: NCT01159288) have evaluated the safety and ability of autologous dendritic cell-derived exosomes, loaded with tumor antigens, to activate tumor-specific cytotoxic T cells in cancer patients [46]. There are also active trials with unmodified stem or progenitor cells EVs. For example, an early phase-1 clinical trial using adipose derived stem cell exosomes in treatment for periodontitis is now recruiting patients for safety evaluation (ClinicalTrials.gov Identifier: NCT4270006). A position paper from the International Society of Extracellular Vesicle (ISEV) highlighted safety and regulatory aspects that must be considered for pharmaceutical manufacturing and clinical application [47].

A concrete future of EVs application in therapy seems not so far away. The scientific community is moving fast, but for a future effective and safe translation of EVs-based therapies into clinical practice, a high level of cooperation between researchers, clinicians and competent authorities should not be neglected.

## 4. Conclusions

In the end, we hope the reviews and articles presented in this Special Issue of Biology will help to shed light on the relevance of extracellular vesicles in different human diseases. Steady research on the components and functions of extracellular vesicles would facilitate the use of extracellular vesicles in diagnostics and therapeutics. Finally, we appreciate the efforts of all contributors to this Special Issue.
